# Non-Invasive Determination of the Mass Flow Rate for Particulate Solids Using Microwaves

**DOI:** 10.3390/s23249821

**Published:** 2023-12-14

**Authors:** Amrit Zoad, Alexander Koelpin, Andreas Penirschke

**Affiliations:** 1 High Frequency Technology, University of Applied Sciences Mittelhessen, 61169 Friedberg, Germany; andreas.penirschke@iem.thm.de; 2 Institute of High-Frequency Technology, Hamburg University of Technology, 21073 Hamburg, Germany; alexander.koelpin@tuhh.de

**Keywords:** mass flow rate, measurement technique, solid concentration determination, non-invasive sensor, velocity detection

## Abstract

This paper presents a novel technique for the mass flow rate determination of particulate solids called the “Sliding Mass Technique”. The mass flow rate is a measure of the mass of a substance that passes through a given cross-sectional area per unit time. Its calculation requires simultaneous detection of the concentration and velocity of the Material Under Test. A novel measurement technique is designed for determining the concentration of the mass flow without the necessity for density evaluation. The mass flow rate is determined by fusing the established concentration results with velocity results obtained from “Microwave Spatial Filtering Velocimetry”. A new metamaterial-based mass flow sensor for particulate solids was designed, realized and measured in an industrial environment. A Software-Defined Radio (Ettus Research^™^’s USRP B210) was utilized as a sensor electronic system for DAQ purposes. A MATLAB app was developed to operate the SDR. Measurements were carried out on-site using a state-of-the-art wood pellet heating system with wood pellets with different moisture contents. The measurement results were found to be in very good agreement with the expected results, which strengthens the feasibility of this newly proposed measurement technique.

## 1. Introduction

Measuring the mass flow of particulate solids down to powder size is an ongoing area of research. Many sectors, such as food processing, wood processing, pharmaceuticals, and surface finishing industries, could benefit immensely from an improved efficiency, output, and quality of their final products. Various methods are available to detect the mass flow, including mechanical [1], electrostatic [2], ultrasonic [3], and optical [4] techniques and more. This study focuses on microwave-based flow sensors only.

A comprehensive investigation of these detection techniques reveals a common goal: a balance of precision and non-invasive intervention. As industries mature, they demand detectors that ensure the integrity of the conveying medium and the conveyed materials without compromising their accuracy. The focus is not only on the development of such non-invasive sensors, but also on measurement techniques that can accurately detect such impairments.

However, non-invasive determination of mass flow, especially for solids, is associated with many complexities. Coriolis meters offer an accurate flow estimation but are only viable for liquids, gases [5], and powders [6]. The added structural discontinuity might become too bulky and even hinder flow for larger hose diameters. On the other hand, venturi tube meters can be used to measure solid and gas flow rates at lower solid loadings if the solid particle size distribution is accurately known [7]. However, this also comes with invasiveness and high costs [8]. There is a clear lack of non-invasive, solid-tailored mass flow sensors.

The mass flow rate, $\dot{m}$, is the measure of the movement of mass, *m*, per unit of time, *t*.

(1) $$\dot{m}=\lim_{\Delta t\to 0}\frac{\Delta m}{\Delta t}=\frac{dm}{dt}$$

**Figure 1:**
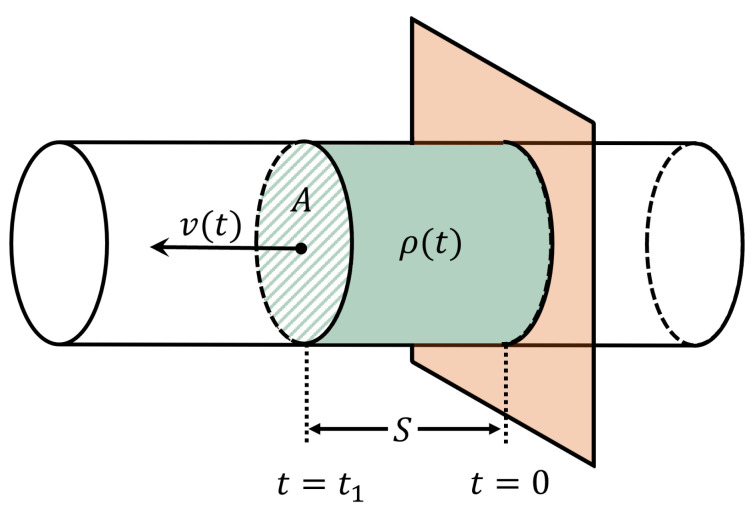
Material Under Test (MUT) flowing through a hose starting from time *t* = 0 to *t* = $t_{1}$.

Figure 1 shows a material of density ρ(t) flowing inside a circular hose of cross-section area *A* at a velocity of v(t). The material has been displaced by a displacement *S* in time interval $t_{1}$. The instantaneous velocity is defined as the displacement per unit time.

(2) $$v\left(t\right)=\frac{S}{t_{1}}$$

The density ρ(t) is defined as the mass per unit volume.

(3) $$\rho \left(t\right)=\frac{m}{S\;\cdot \;A}$$

From Figure 1, the mass flow rate can be defined as the quantity of mass passing through a plane that is placed perpendicular to the direction of the flow per unit time. The formula for the mass flow rate of particulate solids, liquids, and gases is traditionally derived from the volumetric flow rate, $\dot{V}$ [8], given by:

(4) $$\dot{V}\left(t\right)=v\left(t\right)\;\cdot \;A$$

where *v* and *A* are the flow rate or velocity and the cross-section area, respectively.

(5) $$\dot{m}\left(t\right)=v\left(t\right)\;\cdot \;A\;\cdot \;\rho \left(t\right)$$

where ρ is the density.

The density of the mass flow can easily be determined in liquid and gas flow sensors, either with invasive methods like fitting rotors or pressure sensors inside the hose itself or with non-invasive Coriolis flow meters, where the density is determined by the change in the oscillation frequency of the vibrating pipe caused by the flowing fluid. For most sensors, like head loss meters that are meant for liquids and gases, the density is determined from the change in pressure. The change in pressure levels is translated into density and further into mass flow values [8]. In the case of flow sensors designed for solids, such conveniences are not feasible due to the challenging nature of determining density in non-fluidic flows.

There is a direct correlation between the density and mass of a substance in theory, but for practical applications, the density of flowing solids is much harder to determine than for flowing gases or liquids. For example, the material distribution changes from a homogeneous distribution to a highly inhomogeneous distribution up to roping flows due to the flow rate or loading within a pipeline [9]. For microwave- or acoustic-based sensors, one correlation that can be obtained from the solid mass flow is the attenuation in the received signal versus the transmitted signal, as mentioned in [3,10]. The attenuated signal is a function of the mass flow’s density. The denser the flow, the more lossy the amplitude signal appears [10]. One goal of this work is to find a relation between the concentration of the flow and the amplitude change of the received signal in order to derive a new method for determining the mass flow of particulate solids with compact sensors.

The proposed mass flow determination technique is based on the change in the magnitude of the sensor’s transmission factor, $|S_{21}|$. The concentration and velocity of the Material Under Test (MUT) are calculated separately with the newly proposed Sliding Mass Technique and Microwave Spatial Filtering Velocimetry, respectively, and the results are merged at the end.

## 2. Mass Flow Sensor Design

**Figure 2:**
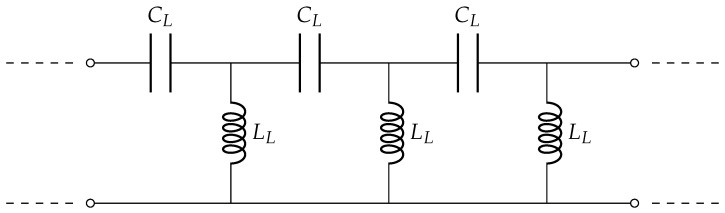
Periodic metamaterial structure: dual of a conventional transmission line equivalent circuit.

The mass flow sensor was intended to be developed using metamaterial technology. Sensors based on metamaterials offer advantages over conventional ones, such as a reduced size and an increased sensitivity [11,12]. An equivalent circuit of a metamaterial structure is shown in Figure 2. In designing the metamaterial-based sensor, several contemporary designs were evaluated. The operation of metamaterial-based sensors typically falls into one of three categories: metasurfaces [13], metamaterial antennas [14], or metamaterial couplers [15]. To identify the most suitable design for mass flow determination, other design goals were taken into consideration.

Given the sensor’s intended application in environments with combustible dust, such as wood pellets under confinement, it is crucial to prevent dust explosions. Such explosions can occur when combustible materials are confined, and an electrostatic discharge (ESD) acts as the trigger. Mitigating this risk is possible by shorting the excitation port within the mass flow sensor. Typically, inductive coupling is employed for this purpose, which involves grounding the inner conductor of the excitation ports. This effectively prevents any DC signal interference with the dust flow and eliminates the risk of an ESD. Consequently, this requirement limits the design choice to a metamaterial coupler, as inductive coupling cannot be implemented in metamaterial antennas or metasurfaces.

Another consideration in designing the sensor is its resonance mode. The mass flow rate is calculated through density measurements, as the density of a pure dielectric material is directly proportional to its dielectric constant or relative permittivity. Pure dielectric materials are detectable by E-fields only. For velocity detection using Microwave Spatial Filtering Velocimetry in the direction of the material flow, in the longitudinal direction at least two electric field maxima are required [16]. Additionally, the operational mode should be close to the sensor’s fundamental mode of the waveguide section to prevent ambiguity and facilitate the design of sensor electronics. Upon designing and conducting EM simulations with CST Microwave Studio, the sensor’s operational mode was determined to be the $TM_{011}$ mode, which has two E-field maxima in the direction of the wood pellet flow. The coupler promotes the propagation of the $TM_{011}$ mode and at the same time prevents other excitation modes.

**Figure 3:**
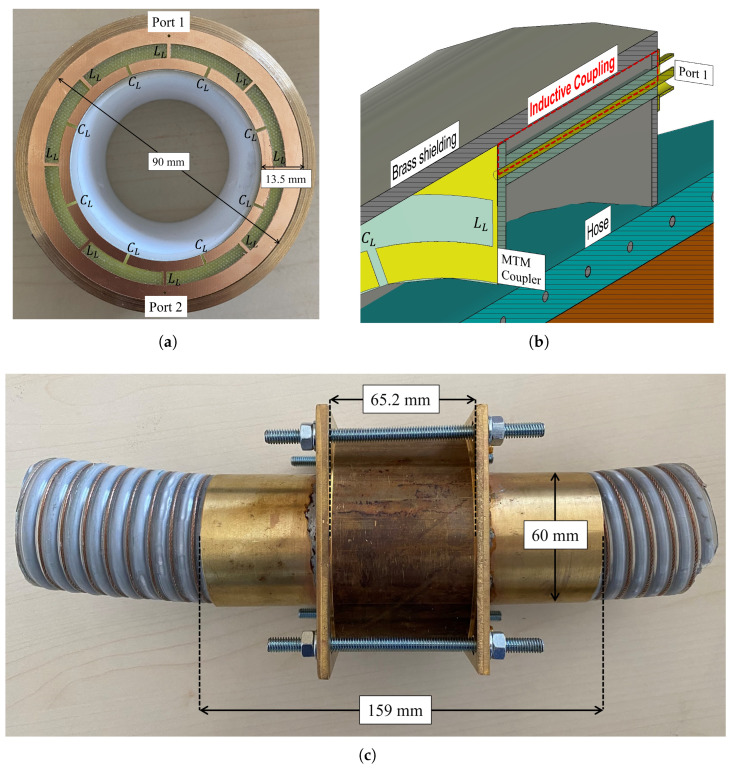
Metamaterial-based mass flow sensor in assembled and disassembled form and its highlighted inductive coupling. (**a**) FR-4-based metamaterial coupler with brass shielding and PTFE support during sensor construction. (**b**) Longitudinal cut of sensor shown in CST Microwave Studio. Inductive coupling shown in red. (**c**) Fully designed mass flow sensor with inserted hose.

**Figure 4:**
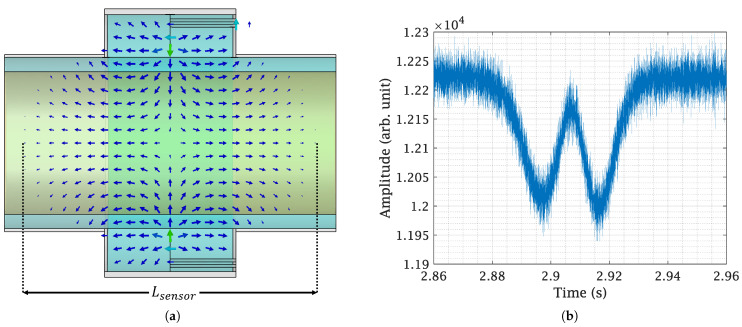
Mass flow sensor’s $TM_{011}$ mode and its significance. (**a**) Sensor’s $TM_{011}$ mode and the extent that it determines the sensor’s effective length, $L_{sensor}$. (**b**) A fluctuation caused by the interaction of a single wood pellet with both E-field maxima of the $TM_{011}$ mode when dropped at free fall.

The designed coupler along with the fully constructed sensor is shown in Figure 3. An equivalency can be seen in the periodic lumped-element model of Figure 2 to the designed coupler in Figure 3a. With CST Microwave Studio EM simulations, $L_{L}$ and $C_{L}$ are determined to be 893.6 nH and 76.63 pF. The coupler design was inspired from a metamaterial-based liner meant for miniaturization of a circular waveguide [17]. The length of a unit cell is 27.87 mm, which is less than one-fifth of the wavelength for a resonance frequency of 2.09 GHz. The coupler was designed on an FR-4 substrate, a glass-reinforced epoxy laminate material. The outer shielding of ports 1 and 2 is soldered to the brass housing of the sensor and the inner conductor is soldered the coupler structure, which in turn connects to the outer part of the sensor, closing the circuit for inductive coupling, as shown in Figure 3b. Finally, the fully assembled sensor with the hose inserted is shown in Figure 3c. The sensor’s operation frequency, $TM_{011}$ mode, and its subsequent impact on a single wood pellet under free fall are shown in Figure 4. When falling, the wood pellet interacts with both the E-field maxima, creating two dips or fluctuations in the magnitude of the sensor’s transmission coefficient ($|S_{21}|$), as shown in Figure 4b. Fluctuations were observed at the sensor’s $TM_{011}$ frequency in continuous wave mode.

## 3. Mass Flow Measurement Overview

**Figure 5:**
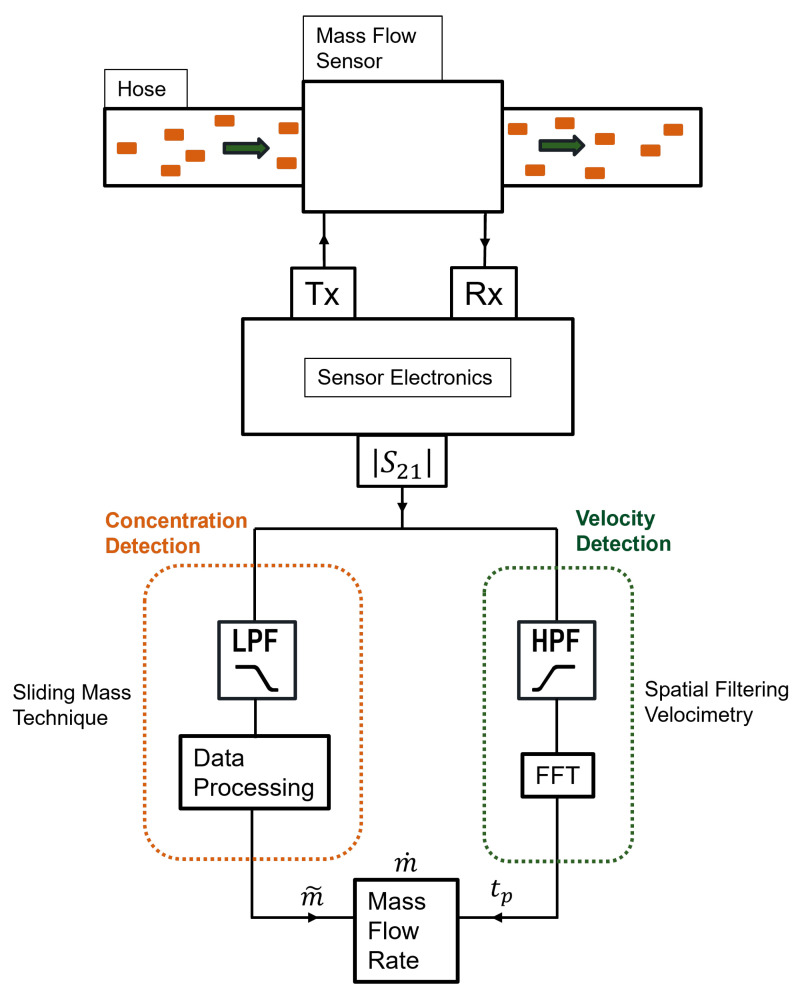
Block diagram of the mass flow process.

The mass flow measurements are, as the name implies, a merging of mass measurements and flow measurements. The mass measurements determine the concentration of the mass flowing through the conveying hose and the flow measurements signify the velocity or the “per time” quantity of the flowing mass. The block diagram in Figure 5 shows how the mass flow is calculated. The detector is connected to the sensor electronics that are responsible for transmission and reception of a continuous wave (CW) signal at the sensor’s resonance frequency. This activates the sensor to operate in the $TM_{011}$ mode. After the sensor electronics transmission and reception channels initialize at the resonance frequency, the conveying process can be started. The flowing mass alters the transmitted signal’s amplitude and an attenuated signal containing the mass flow information is received at the sensor system’s receiving end. These data are processed in two branches. Data processing is performed only on the signal’s amplitude. The left branch processes the velocity and the right branch processes the concentration. The velocity is determined by the Microwave Spatial Filtering Velocimetry technique and the concentration is obtained by a newly proposed method explained in this paper called the “Sliding Mass Technique”. Both are combined at the end and the mass flow rate is determined.

## 4. Sensor Electronics: USRP B210

**Figure 6:**
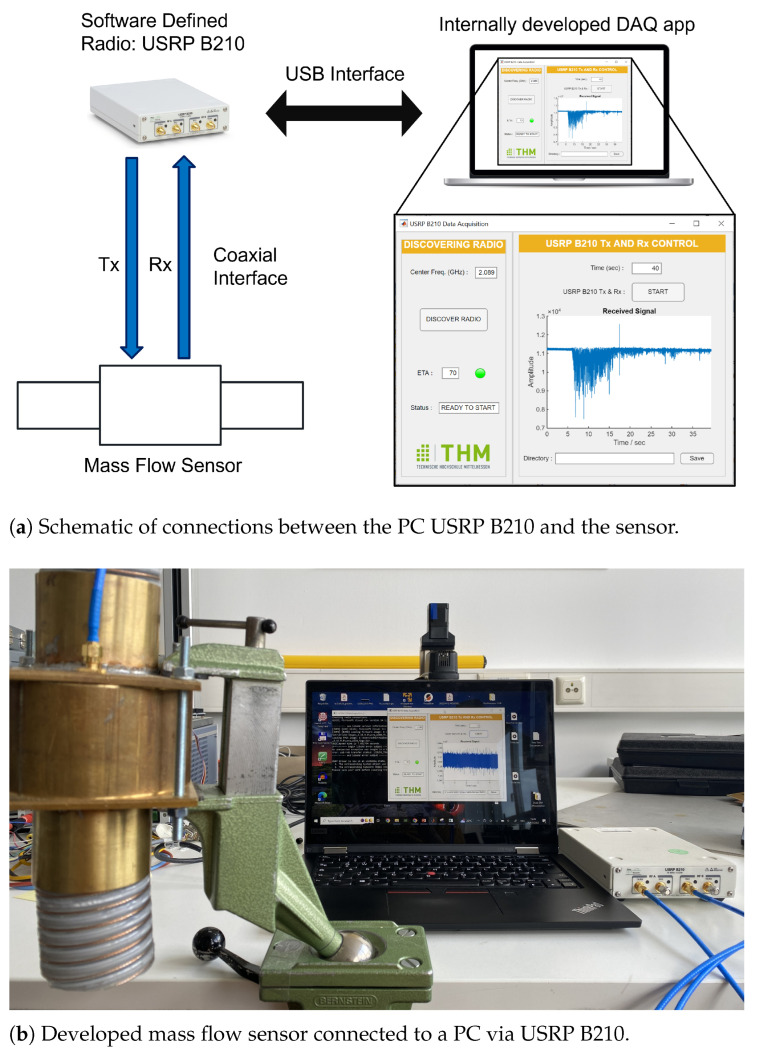
Setup of mass flow sensor and electronics.

The role of the sensor electronics is to operate the sensor in the desired mode and perform data acquisition. For this purpose, Ettus Research’s Software-Defined Radio (SDR) USRP B210 was utilized. Figure 6a shows a schematic representation of the setup, while Figure 6b shows its practical equivalence. Both figures show an app that was developed internally for DAQ purposes with the help of MATLAB App Designer. The app enables the user to directly control the Software-Defined Radio USRP B210 via a USB interface. The SDR’s Tx and Rx channels are connected to the sensor via coaxial cables. Both channels are simultaneously switched ON and a CW signal with the desired frequency is sent from the Tx channel that passes through the sensor and is received at the Rx channel. This received signal is stored in a file for further processing.

## 5. Sliding Mass Technique (SMT): Proposed Concentration Estimation Method

**Figure 7:**
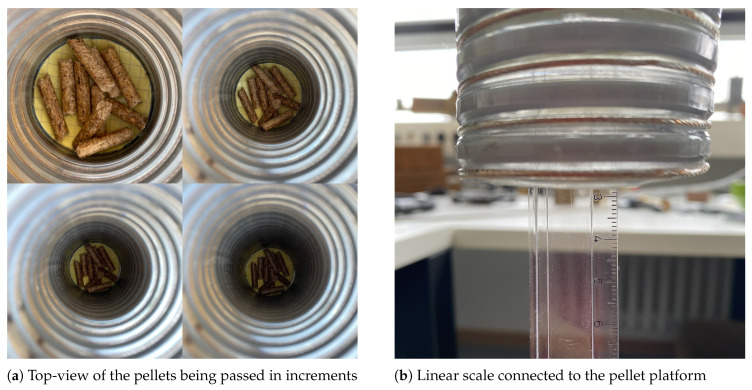
Measurement setup of the mass flow sensor for the proposed Sliding Mass Technique.

**Figure 8:**
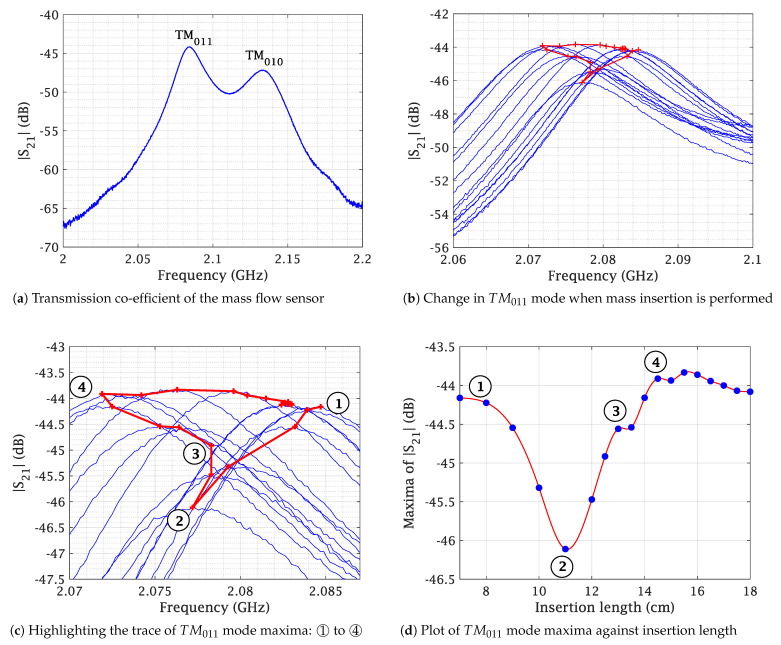
(**a**) Sensor performance and (**b**–**d**) results when 10 g of sample mass is passed in increments (from ➀ to ➃) for the proposed Sliding Mass Technique.

**Figure 9:**
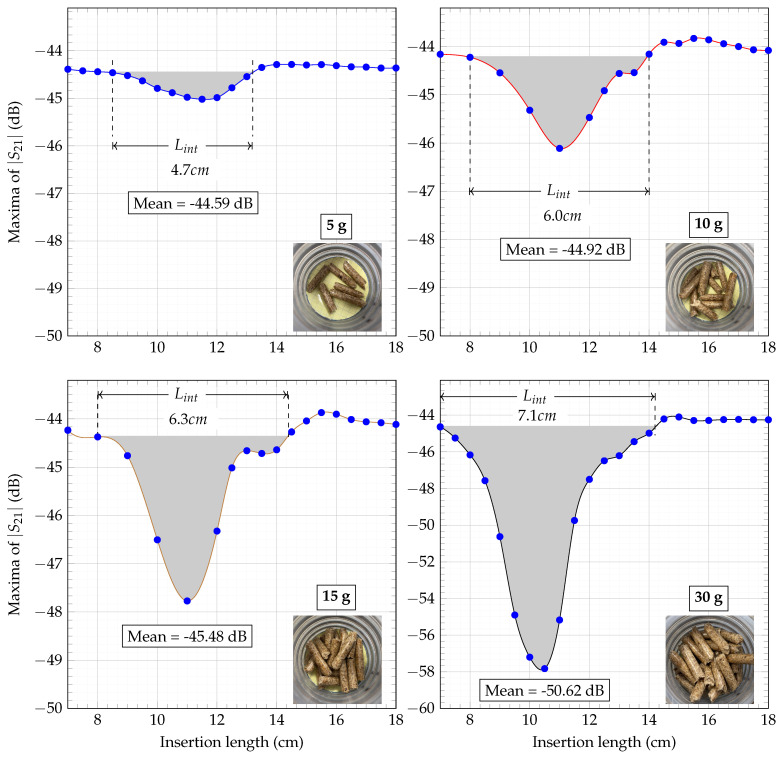
Trace of the movement of the $TM_{011}$ mode maxima against the insertion length for 5 g, 10 g, 15 g, and 30 g mass samples.

**Figure 10:**
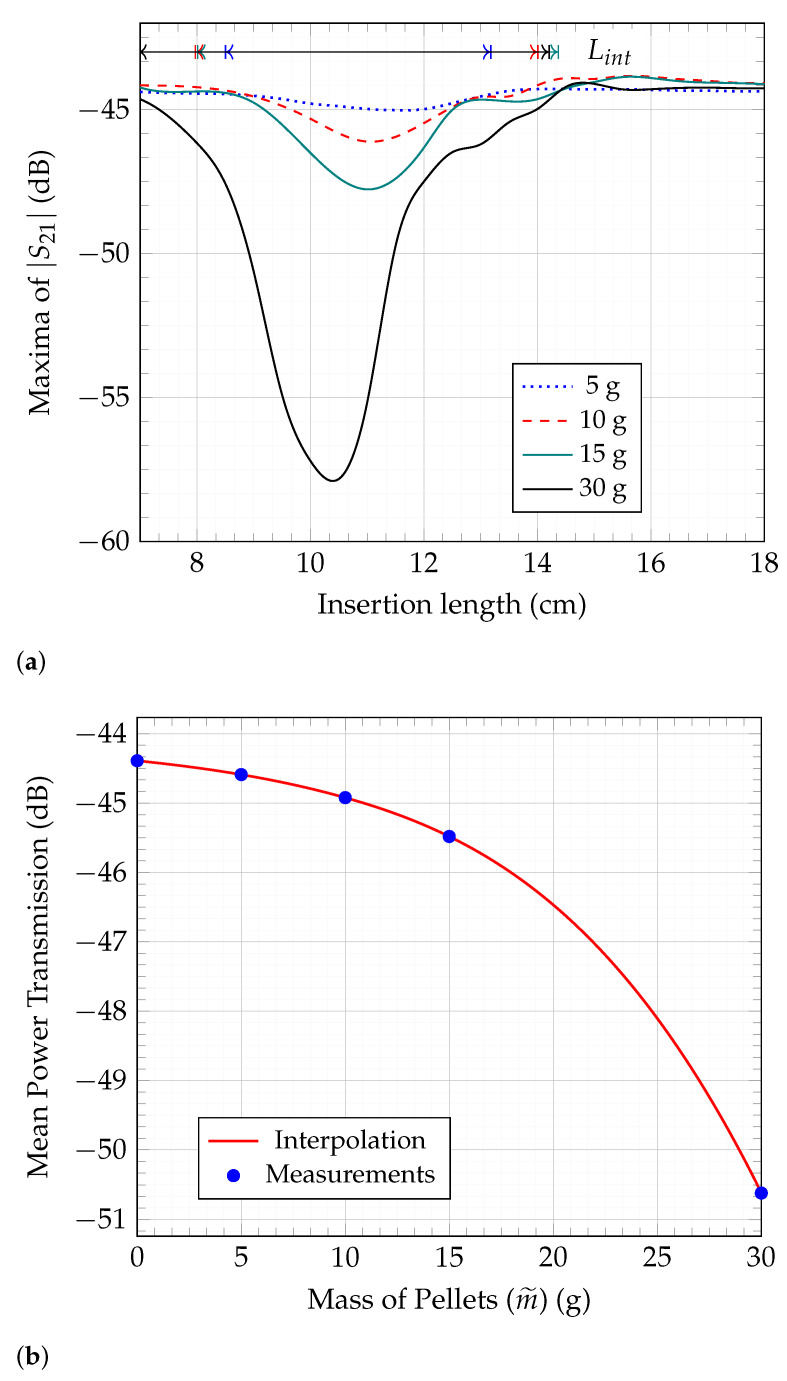
Average values of $|S_{21}|$ for the interaction length were realized from (**a**) and plotted as points (blue) in (**b**) and interpolated (red). (**a**) Compilation of plots from Figure 9 to showcase changing $L_{int}$. (**b**) Relationship of mass of pellets and their impact on the mean transmitted power when pass through the designed sensor.

To determine the mass flow in a system, it must first be divided into its individual components, namely concentration and velocity. The proposed SMT method focuses exclusively on the concentration aspect, effectively isolating it by eliminating the velocity component of the mass flow. This is achieved through the following steps:

Step 1: The process begins by attaching the sensor to the hose to emulate a practical scenario. The sensor is then connected to a calibrated Vector Network Analyzer (VNA). The Material Under Test (MUT) with a known mass is placed on a platform linked to a linear scale. This scale, inserted through the sensor in small increments of known lengths, records the shift in the transmission S-parameter, $S_{21}$, with each increment. This procedure continues from the point the MUT enters the hose until it has completely passed through. This process is illustrated in Figure 7.
Step 2: Both sensor modes, $TM_{011}$ and $TM_{010}$, are depicted in Figure 8a. Of these, only the $TM_{011}$ mode is used due to its characteristic of having two E-field maxima. The measurement results for a 10 g wood pellet sample are presented in Figure 8b–d. The 10 g sample, placed on a platform, was slid through the sensor while noting the insertion length, as described in step 1. Changes in $|S_{21}|$ during the mass’s movement are shown in Figure 8b, where the red trace indicates the path of the maxima throughout the process. This trace is further highlighted in Figure 8c. The path is marked with numbers from ➀ to ➃. In Figure 8d, the maxima of $|S_{21}|$ from each slide are plotted in blue against their respective insertion lengths and interpolated in red.
Step 3: The previous step was repeated for multiple mass samples, and the $|S_{21}|$ maxima were plotted against the insertion length in Figure 9, similar to Figure 8d. In Figure 4a, the effective length of the sensor, $L_{sensor}$, is shown, defined by the extent of the E-fields of the $TM_{011}$ mode. It was observed that the sliding of the mass samples added an additional length, Δl, to it. This increase is attributable to the height of the wood pellet sample used in these measurements. As the quantity of the sample increased, so did its height, causing earlier interaction with both E-fields on the insertion length axis. Thus, Δl is equivalent to twice the height of the mass sample for the $TM_{011}$ mode, resembling the slug flow regime in practice, characterized by periods of little to no movement, followed by the rapid passage of a large chunk of material. Acknowledging these unique flow dynamics, we define the ’interaction length’ as the cumulative distance encompassing both $L_{sensor}$ and Δl, providing a comprehensive measurement that accounts for the entire span of material interaction within the system. The interaction length is given by $L_{int}=L_{sensor}+\Delta l$. The mean values of the $|S_{21}|$ maxima are calculated over the extent of $L_{int}$. The greater the mass quantity, the longer $L_{int}$ and the deeper the curve. The interaction length, $L_{int}$, and the corresponding mean of this length are shown in Figure 9 for 5 g, 10 g, 15 g, and 30 g mass samples. It should be noted that the estimation of the extent of $L_{int}$ and thus the mean of $|S_{21}|$ maxima are determined by drawing a line parallel to the x-axis that intersects the curve on either side of the dip at the highest y-axis value. The region under this line is greyed out for clear visibility in Figure 9. For the purpose of comparison, all plots from Figure 9 are superimposed in Figure 10a.
Step 4: The mean value of each mass sample from the previous step is plotted against its corresponding sample mass, and an interpolation curve is generated that passes through these points, as shown in Figure 10b. It is noteworthy that this curve is directly proportional to the relative permittivity of the MUT. This establishes a relationship between the mean power absorption and the MUT’s mass. The mass quantity derived from this relationship will include the interaction length, $L_{int}$. Consequently, it can be defined as the mass of the flowing MUT as observed from the limited perspective of a specific mass flow sensor. Due to this specificity, it is denoted by $\tilde{m}$ and is relevant only to the sensor it was measured with. Thus, different $\tilde{m}$ values may exist for the same wood pellet sample across different mass flow sensors. The interpolated curve from Figure 10b is normalized with the sensor system transmitter power, and the data received are matched to the corresponding $\tilde{m}$ values to determine the concentration.

## 6. Microwave Spatial Filtering Velocimetry: Velocity Determination

**Figure 11:**
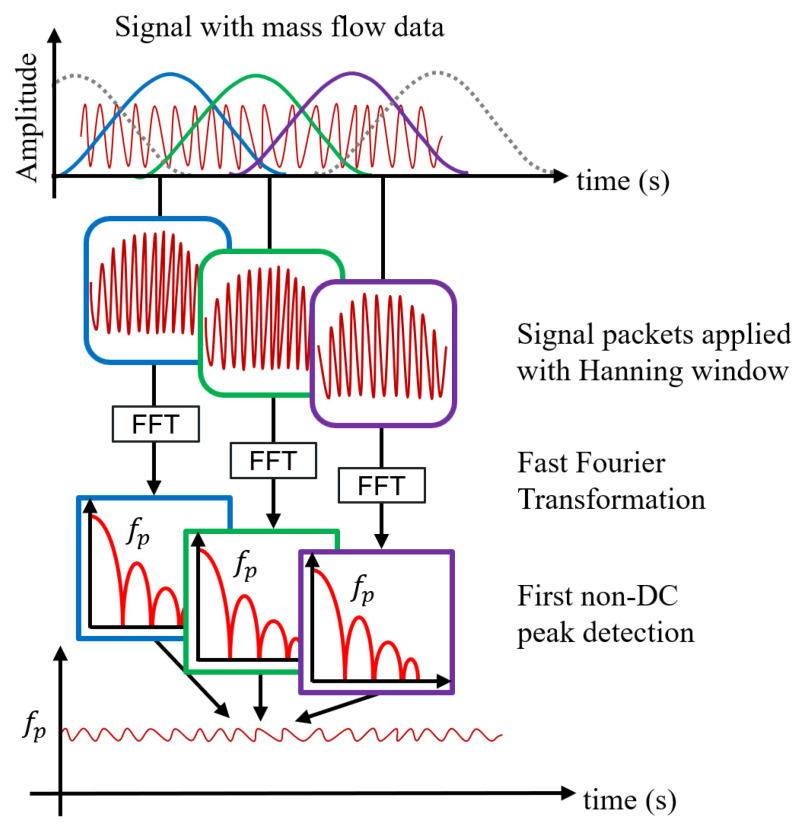
Block diagram of Microwave Spatial Filtering Velocimetry process.

The Microwave Spatial Filtering Velocimetry (μWSFV) process is used to determine the velocity of the mass flow. The block diagram of the entire process is depicted in Figure 11. Initially, the signal containing mass flow data is divided into smaller, equally sized overlapping packets. A Hanning window is applied to each packet to reduce edge artifacts. Overlapping is utilized to prevent loss of edge data and to enhance the resolution of the processed signal. Subsequently, the Fast Fourier Transformation (FFT) is applied to each processed packet, converting the data from the time domain to the frequency domain. In this transformed data, the velocity information is found in the first non-DC peak, denoted by $f_{p}$. For the proposed SMT, this is converted into a time quantity, $t_{p}$, which is given by,

(6) $$t_{p}=\frac{1}{f_{p}}$$

The velocity of the flow for a sensor with *k* number of E-field maxima and minima can be calculated from [16] as:

(7) $$v_{mut}=\frac{f_{p}}{2}\;\cdot \;\frac{L_{sensor}}{k}$$

## 7. Mass Flow Rate Determination: Combining SMT and μWSFV

For concentration determination, the left branch of the block diagram in Figure 5 is followed. The data received at the sensor system’s receiver end are processed in packets and the mean value of each packet is directly correlated with the empirical data from Figure 10 to determine the mass of the MUT, $\tilde{m}$(t). $\tilde{m}$(t) is a concentration parameter that takes into consideration the changing $L_{int}$. It is a measure of the instantaneous mass of the MUT flowing in the measurement section.

To determine the mass flow rate (g/s), the concentration parameter $\tilde{m}$(t) is divided by the velocity-related per-time quantity obtained from μWSFV, $t_{p}\left(t\right)$, found from Figure 11 and Equation (6).

The concentration of the flow, $\tilde{m}\left(t\right)$, is determined from the “Sliding Mass Technique” and the per-time quantity or velocity of the flow, $t_{p}\left(t\right)$, is derived from the “Microwave Spatial Filtering Velocimetry”. They are combined together to give the newly derived formula for the mass flow rate for solids:

(8) $$\dot{m}\left(t\right)=\frac{\tilde{m}\left(t\right)}{t_{p}\left(t\right)}$$

It should be taken into consideration here that since both $\tilde{m}$ and $t_{p}$ are a function of time, they can be plotted individually against the time period for which the measurements were taken, albeit with little meaning.

## 8. Materials and Methods

MATLAB App Designer with “Communications Toolbox Support Package for USRP Radio” was used for developing and prototyping the internal DAQ app shown in Figure 6.

The MATLAB function “spline” was used for data interpolation in Figure 8d and Figure 9.

**Figure 12:**
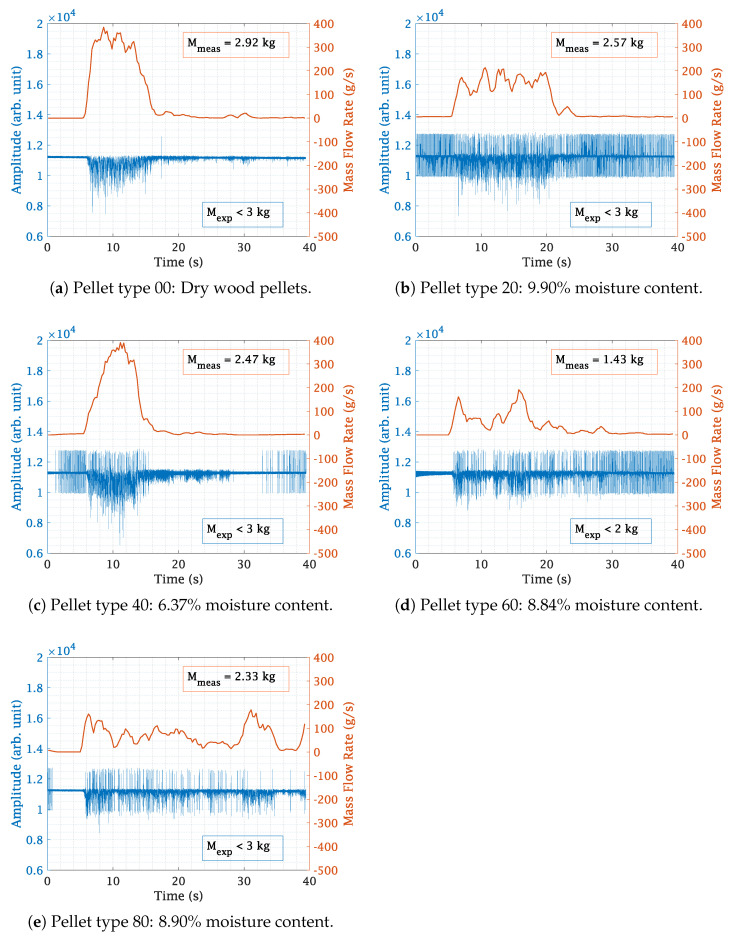
Processed mass flow data obtained with the proposed SMT and μWSFV.

The MATLAB functions “lowpass” and “smooth” were utilized for removing noise and smoothening the final mass flow rate plot in Figure 12a–e.

The total mass in Figure 12a–e was calculated from the area under the curve with the “trapz” MATLAB function.

## 9. Measurement Results and Discussion

**Figure 13:**
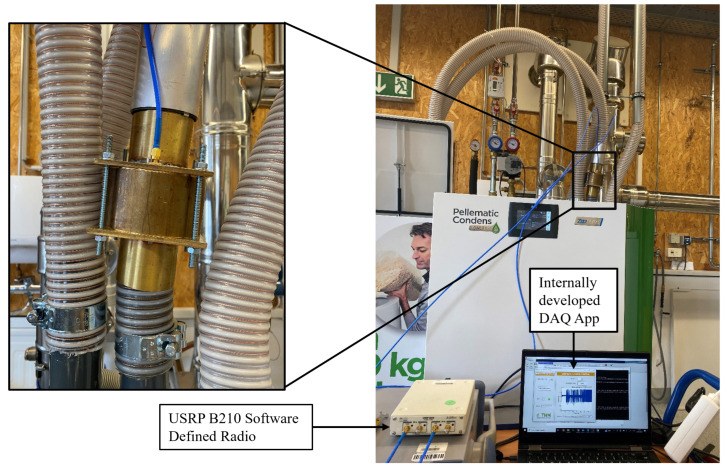
Mass flow measurement setup showcasing the mass flow sensor, USRP B210, DAQ app, and the wood pellet heating system.

The measurement results were obtained using an actual wood pellet heating system, as depicted in Figure 13. To ensure diversity in the measurements, pellets with varying moisture content levels were used. The objective was to determine how different moisture content percentages in wood pellets affect the mass flow sensor readings. The wood pellets were weighed before being placed in the container. A vacuum pump conveyed the wood pellets from the container to the heating system through the designed mass flow sensor. Since the weight of the wood pellets was already known, the proposed mass flow rate determination technique was employed to measure both the continuous mass flow rate and the total mass flow. The expected, $M_{exp}$, and measured, $M_{meas}$, results are presented in Figure 12a–e. The $M_{exp}$ results represent a known quantity: the mass of wood pellets as measured by a weighing scale before being put to test. Meanwhile, the $M_{meas}$ results refer to the quantity calculated by the novel technique proposed in this paper, specifically the area under the mass flow rate curve. The use of a less than sign for the $M_{exp}$ results is due to the fact that the vacuum pump was unable to convey all the pellets, leaving some quantity in the container after the measurements were taken.

For all measurements, the vacuum pump was turned ON after 5 s from the start of sensing, as evident in Figure 12a–e. In Figure 12b, the zero floor appears slightly elevated, likely due to a calibration error in the SDR, possibly causing an increase in Tx or Rx gain from its predefined value. Figure 12c,e also exhibit a slight non-zero value in the first 5 s, which seems like an error from the SDR. In Figure 12a, unaltered dry pellets were used, showing a close match of the $M_{meas}$ results at 2.92 kg to the $M_{exp}$ results, projected to be less than 3 kg.

The presence of noise in most measurements is notable; however, it does not impact the mass flow rate results, as the concentration and velocity were determined independently. Further investigation of these noise contributions is necessary to fully comprehend their impact and origins.

The flow type can also be inferred from the plots. In Figure 12a,c, the vacuum pump conveyed almost all the pellets at once between 5 s and 17 s. In Figure 12b,d, this period is extended, spanning from 5 s to 25 s. In contrast, Figure 12e shows constant pellet conveyance by the pump until the end.

The discrepancy between the $M_{meas}$ and $M_{exp}$ mass flow results may arise from several factors, including potential sensor system calibration errors, the vacuum pump’s limited capacity to transport the entire mass within the specified timeframe, the moisture content in the wood pellets, etc. Despite these factors, the results show a strong correlation between the $M_{meas}$ and $M_{exp}$ values. Measurements should be carried out at a greater number of points in Figure 9 to determine the nature of the curves more accurately, thereby increasing the accuracy of the results. When superimposed on one another in Figure 10, it was observed that the 30 g results were shifted to the left side. This could be due to a human error or any other factor. However, it does not create problems for the mean calculations. To ensure accurate results, the measurements need to be repeated several times to verify the reliability of these results. Calibration errors in sensor electronics can occur due to changes in temperature or humidity in the air. Additionally, there can be changes in the sensor’s resonance frequency, which directly impact its accuracy. To address these issues, the use of commercially available phase-locked loops with auto-calibration features could prove beneficial. Further measurements accounting for the mass of pellets remaining in the vacuum pump chamber are required. Additionally, de-embedding the effects from the platform and the linear scale used in the sliding process should enhance accuracy. For this, results obtained from an empty platform slid through the hose, as shown in Figure 9, should be subtracted from the measurements. Wood pellets are prone to moisture absorption, and the unused modes of the sensor that are multiples of the resonance frequency could be utilized for precise moisture measurements, as shown in [18]. The sensitivity and detection limits of the sensor need to be further examined. Figure 4b shows that the sensor can detect a single wood pellet, implying no minimum limit. However, since the detection is based on attenuation of the transmitted signal, there will be an upper threshold limit of the wood pellet concentration, beyond which, the sensor’s output signal will be too low to be detected anymore.

Table 1 offers a comparative overview of the proposed sensor against contemporary ones, detailing their features and measurement techniques. The table starts by identifying the purpose of each sensor, focusing on those used for similar applications, such as solids/gas flow. It then describes the operating principles, including microwave (coupler and antenna), acoustic, electrostatic, ultrasonic, and other types. The methodologies for determining velocity and concentration in mass flow measurements are also discussed. The table evaluates whether the sensors are non-invasive, a feature crucial for measuring the mass flow rate without disrupting the flow of the Material Under Test (MUT). It also considers the safety features, such as ESD protection, relevant only for microwave-based sensors.

## 10. Conclusions and Outlook

A state-of-the-art metamaterial-based mass flow sensor was designed and yielded a performance in line with expectations. The Sliding Mass Technique, an amplitude-based, non-invasive method for determining concentration without establishing density measurements, was effectively showcased. Velocity assessments were independently conducted using Microwave Spatial Filtering Velocimetry, with the data being subsequently processed to ascertain the mass flow rate. Field measurements were executed and were observed to align with anticipated outcomes. While a metamaterial-based mass flow sensor was employed for this particular demonstration, the results are replicable using conventional sensor designs, showcasing the versatility and robustness of the findings.

The prospective sensor can replace most commercially available mass flow sensors. Some conveying hoses, like that in the wood pellet heating system, have earthing wires built-in and therefore they cannot be altered and require a non-invasive solution for mass flow detection. The proposed sensor is especially designed for such use cases. For this specific application, no dedicated sensor has existed up to now.

## Figures and Tables

**Table 1:** Comparison with contemporary mass flow sensors, their features and measurement techniques.

	This Work	[19]	[20]	[10]	[3]	[4]	[21]	[16]
Purpose	Solids/gases	Solids/gases	Solids/gases	Pulverised solids/gases	Solids/gases	Solids/gases	Solids/gases	Solids/gases
Operating principle	Microwave coupler	Acoustic and Electrostatic	MEMS and Capacitive	Microwave antenna	Elbow and Ultrasonic	Acoustic	Electrostatic	Microwave coupler
Velocity Detection	Microwave Spatial Filtering Velocimetry	Multiple channel cross-correlation	Coriolis: Direct Mass Flow Detection	Multiple channel cross-correlation	Force Vortex	Multiple channel cross-correlation	Waveform Charact-eristics in Time Domain	Microwave Spatial Filtering Velocimetry
Concen-tration-Detection	Sliding Mass Technique	Collision with Electrode		Concentr-ation meter	Pressure difference	Cross-correlation	Collision with Electrode	Phase Shift
Non-Invasive Sensing	Yes	No	Yes	Yes	No	No	No	Yes
Number of sensing elements	1	2	1	6	2	1	2	2
Grounding for ESD Protection	Yes	No	N.A.	No	N.A.	N.A.	No	No

## Data Availability

Data are contained within the article.

## Abbreviations

The following abbreviations are used in this manuscript:

MUT	Material Under Test
USRP	Universal Software Radio Peripheral
SDR	Software-Defined Radio
SMT	Sliding Mass Technique
μWSFV	Microwave Spatial Filtering Velocimetry
DAQ	Data Acquisition
Tx	Transmitter
Rx	Receiver
